# Evidence for a histaminergic input from the ventral tuberomammillary nucleus to the solitary tract nucleus involved in arterial pressure regulation

**DOI:** 10.14814/phy2.13095

**Published:** 2017-03-14

**Authors:** Ko Yamanaka, Sabine S. Gouraud, Miwa Takagishi, Akira Kohsaka, Masanobu Maeda, Hidefumi Waki

**Affiliations:** ^1^ Department of PhysiologySchool of Health and Sports ScienceJuntendo UniversityChibaJapan; ^2^ Department of BiologyFaculty of ScienceOchanomizu UniversityTokyoJapan; ^3^ Department of Therapeutic Health PromotionKansai University of Health SciencesOsakaJapan; ^4^ Department of PhysiologyWakayama Medical University School of MedicineWakayamaJapan; ^5^ Department of PhysiologyGraduate School of Health and Sports ScienceJuntendo UniversityChibaJapan

**Keywords:** Arterial pressure, histamine, nucleus tractus solitarius, tuberomammillary nucleus

## Abstract

The tuberomammillary nucleus (TMN) of the posterior hypothalamus has a high density of histaminergic neurons, the projection fibers of which are present in many areas of the brain, including the nucleus tractus solitarius (NTS), which controls arterial pressure (AP). In this study, we investigated whether the TMN–NTS pathway is involved in central cardiovascular regulation. Bicuculline, a gamma‐aminobutyric acid type A (GABA_A_) receptor antagonist, was microinjected into the ventral TMN of anesthetized rats and its effects on AP and heart rate (HR) were observed. We also evaluated the effect of cetirizine, an H_1_ receptor antagonist, microinjected into the NTS on cardiovascular responses induced by electrical stimulation of the TMN. Both AP and HR increased following bicuculline microinjection into the ventral TMN. Similar pressor and tachycardic responses were observed after electrical stimulation of the ventral TMN. Microinjection of cetirizine into the NTS partially inhibited the pressor response but had no effect on HR. Finally, the treadmill test was associated with a high level of c‐Fos expression in both ventral TMN and NTS neurons. These results suggest that the TMN–NTS pathway is involved in regulation of AP, presumably under a high‐arousal phase, such as that during exercise.

## Introduction

The nucleus tractus solitarius (NTS) is the central termination site of baroreceptor inputs. On the basis of the information received by NTS neurons, cardiac sympathetic/parasympathetic neurons and vasomotor sympathetic outflows are regulated to stabilize arterial pressure (AP). Thus, the NTS is crucial for maintenance of cardiovascular homeostasis (Barraco 1994; Sapru 2004; Andresen and Paton 2011). It also receives numerous inputs from other central nuclei in the brainstem and from the areas of the hypothalamus which are involved in the cardiovascular defense reaction, such as the paraventricular nucleus and the dorsomedial hypothalamus (Dampney et al. 2008; Michelini and Stern 2009; Dampney 2015). Moreover, direct projections from neurons of the spinal dorsal horn, which are innervated by afferent inputs from skeletal muscle, have been identified in the NTS (Potts et al. 2003; Potts 2006). Thus, the NTS is also a vital brainstem component that has integrative functions and adjusts AP to appropriate levels in response to mental stress and physical activities (Doba and Reis 1973; Talman et al. 1981; Andresen 2004; Sapru 2004; Thrasher 2006). Physical activities, such as exercise, are generally accepted to increase both AP and heart rate (HR) mainly because of sympathoexcitation (Ludbrook and Graham 1985; Miki et al. 2003; Matsukawa 2012; Waki 2012) and NTS is one of the key nuclei involved in cardiovascular control (Michelini and Stern 2009; Waki 2012; Waki et al. 2013).

Considering these neuroanatomical/functional aspects, a variety of neurotransmitters and neuromodulators have been identified within the NTS. One of these is histamine, a type of monoamine neurotransmitter (Haas et al. 2008). Histaminergic receptors have long been identified in the medulla oblongata, including the NTS (Schwartz et al. 1991; Bealer 1999; Bárbara et al. 2002; Poole et al. 2008), and our recent findings have demonstrated that H_1_ receptors are the most dominantly expressed histaminergic receptor in NTS neurons (Bhuiyan et al. 2011; Takagishi et al. 2014). We have also shown that microinjection of either histamine or 2‐pyridylethylamine, an H_1_ receptor‐specific agonist, into the NTS, where baroreceptor‐sensitive neurons are abundantly found, increased AP and HR in a dose‐dependent manner (Bhuiyan et al. 2011; Takagishi et al. 2014). These findings suggest the likely involvement of histamine in the NTS‐mediated central cardiovascular control in response to certain physiological conditions. In addition, central histamine is known to be associated with arousal levels (Haas and Panula 2003; Haas et al. 2008), suggesting its role in maintaining elevated levels of AP and HR seen during a high‐arousal phase, such as during physical activity.

The premise of our study was to pinpoint the specific area of the brain that influences NTS functions via histaminergic neurons. In this regard, the tuberomammillary nucleus (TMN) of the posterior hypothalamus posits as the most suitable candidate since histaminergic neurons are found exclusively in the TMN and fibers extending from these neurons have also been found in the NTS (Bealer 1999; Takagishi et al. 2014). However, till date, there has been no direct evidence to support that the TMN controls the cardiovascular system by modulating NTS neurons. Hence, our study was designed to investigate whether a TMN–NTS pathway was involved in the central regulation of the cardiovascular system under certain physiological conditions.

In this study, we investigated whether the TMN–NTS pathway is involved in the central cardiovascular regulation. We microinjected bicuculline, a GABA_A_ receptor antagonist, into the ventral TMN or electrically stimulated the same brain area. In both cases, we found pressor and tachycardic responses. The electrically induced pressor responses were partially inhibited by cetirizine, an H_1_ receptor antagonist, microinjected into the NTS. We histologically confirmed that the TMN neurons directly project to the NTS. Finally, treadmill exercise, which is considered as a high‐arousal phase, induced c‐Fos expression in the ventral TMN and NTS neurons. These results suggest that the TMN–NTS pathway may be involved in AP regulation, presumably under physical activities, such as exercise.

## Methods

### Animals and animal care

Male Wistar rats (8–12 weeks old, 250–330 g) obtained from either Kiwa Laboratory Animal Company (Wakayama, Japan) or SLC (Shizuoka, Japan) were used in this study. The animals were housed in a temperature‐controlled room with a fixed 12‐h:12‐h light–dark cycle (18:00–06:00 and 06:00–18:00). Food and tap water were given ad libitum. All experiments were approved by the Ethics Committee for Animal Experiments at Wakayama Medical University and Juntendo University and complied with guidelines set by the Physiological Society of Japan.

### Physiological examinations

#### General procedures

Animals were anesthetized using intraperitoneal (i.p.) urethane (1.45 g/kg; Tokyo Kogyo, Tokyo, Japan). Level of anesthesia was monitored regularly by assessing limb withdrawal response to a noxious stimulus (toe pinch), and an additional dose of urethane (0.145 g/kg, i.p.) was administered when necessary. Rectal temperature was monitored and maintained at 37°C with the help of a heating pad (BWT‐100; Bio Research Center, Nagoya, Japan). The trachea was cannulated, and a rodent respirator (SN‐480–7 Shinano Respirator; Shinano Manufacturing, Tokyo, Japan) was used to facilitate artificial breathing. AP and HR were continuously measured by a previously described method using the PowerLab system (PowerLab/8s; ADInstruments, Nagoya, Japan) (Bhuiyan et al. 2011; Takagishi et al. 2014). The femoral veins were cannulated with polyethylene tubes for continuous infusion of physiological saline (0.8 mL/100 g/h) containing the muscle relaxant pancuronium bromide (0.08 mg/kg/h). In experiments where pancuronium was administered, the adequacy of anesthesia was assessed periodically throughout the experiment by observing the AP response to a toe pinch, and supplemental urethane (0.145 g/kg i.p.) was given when necessary.

#### TMN microinjections

Anesthetized rats were placed in a stereotaxic head holder (SR‐5; Narishige Scientific Instrument Lab, Tokyo, Japan). The skull was exposed and a small hole was drilled on the right side. A unilateral microinjection (total volume: 50 nL) of either bicuculline, a GABA_A_ receptor antagonist (bicuculline methiodide, Sigma‐Aldrich, St Louis, MO) at three different doses (100 pmol/50 nL: *n* = 7; 10 pmol/50 nL: *n* = 5; or 1 pmol/50 nL: *n* = 5), or vehicle (saline, *n* = 4) was administered into the ventral TMN (4.1–4.3 mm caudal to bregma, 1.2–1.5 mm lateral to the midline, and 8.6–9.0 mm ventral to the dura). A glass micropipette (GC200F‐10; Harvard Apparatus, Edenbridge, UK) with an outside diameter of 20–30 *μ*m connected to a Hamilton microsyringe mounted on a syringe pump (LEGATO110; KD Scientific, Holliston, MA) was used for the microinjections. The position of the micropipette was also marked by a concomitant injection of 50 nL FluoSpheres (F8811; Thermo Fisher Scientific, MA).

#### TMN stimulation and NTS microinjections

A separate set of study rats (*n* = 6) was used for this experiment. Animals were anesthetized and prepared for electrical stimulation of TMN as previously described. A concentric microelectrode (OA‐212‐053a; Unique Medical, Japan) was vertically inserted into the ventral TMN. For bilateral microinjections of equal volumes (50 nL) of either cetirizine dihydrochloride (TOCRIS Bioscience, Missouri 63021) or vehicle (saline) into the NTS (0.5 mm rostral to the calamus scriptorius, 0.4 mm lateral from the midline, and at a depth of 0.5 mm from the dorsal surface of the brainstem), the caudal dorsal medulla of animals was exposed through a midline incision in the dorsal neck. Details of a glass micropipette and a syringe pump are described in the previous section.

#### Experimental protocols

Unilateral stimulation of TMN was done using biphasic negative–positive electrical pulses (200 *μ*A, 0.5 msec pulse, 50 Hz, and 30 sec duration) at least 30 min after cardiovascular parameters had stabilized following surgical operations. Subsequently, after a recovery period of ≥30 min, cetirizine (100 pmol/50 nL) was bilaterally microinjected into the NTS, and the TMN was electrically stimulated again within 3 min. The effect of vehicle (saline) administration into the NTS was also recorded (*n* = 4). At the end of the experiment, a direct current of 1 mA was applied for 5 sec by the stimulating electrode to make a lesion. The animals were killed by urethane overdose (3 g/kg intravenously), and whole brains were collected, postfixed with 4% paraformaldehyde for at least 48 h, and transferred into phosphate‐buffered saline (PBS) containing 30% sucrose before sectioning (thickness: 40 *μ*m) with a freezing microtome (REM‐710; Yamato Kohki Industrial Co., Saitama, Japan). Finally, the sections were mounted for viewing. Only brain sections from rats that had microstimulation sites precisely in the ventral TMN were used in the analysis.

### Histological examinations

#### c‐Fos detection in the TMN and NTS

To examine whether the TMN is involved in exercise‐related brain activities, expression of the proto‐oncogene product c‐Fos was immunohistochemically detected. Study rats (*n* = 8) were familiarized with exercise by being made to run on a treadmill at 3–25 m/min for a period of 10–30 min every 3 days for duration of 4 weeks prior to the experiments. On the day of the experiment, the animals were divided randomly into control (no‐exercise) and exercise groups. Animals in the exercise group (*n* = 4) were placed on the treadmill and run at 20 m/min for 60 min. Rats in the no‐exercise group (*n* = 4) were placed on the treadmill for 60 min, but the belt was kept stationary. Following this, the animals were returned to their cages for 30 min to recover and to allow enough time for c‐Fos to be expressed in activated regions of the brain. Subsequently, the rats were killed by isoflurane, perfused transcardially with heparinized saline followed by 4% paraformaldehyde, fixed in 4% paraformaldehyde, and brain sections were made as previously described. The sections were rinsed in PBS, placed in 10% serum with 0.3% Triton X‐100 for 15 min at room temperature, rinsed again, and then incubated overnight at 4°C with an anti‐c‐Fos antibody (sc‐52‐G; Santa Cruz Biotechnology, Inc., Santa Cruz, CA; 1:200 dilution in PBS with 1% serum and 0.3% Triton X‐100). On the following day, sections were rinsed in PBS and incubated with biotinylated horse anti‐goat immunoglobulin G (Vector Laboratories, Burlingame, CA; 1:500 dilution) antibody for 1 h. Following yet another rinse, the sections were incubated with streptavidin‐conjugated Alexa‐Fluor 488 (Molecular Probes, Eugene, OR; 1:500 dilution) for 1 h. Finally, the sections were washed in PBS, mounted on VECTASHIELD (Vector Laboratories), and imaged using a fluorescence microscope (CKX41, Olympus, Japan). We mainly focused on the TMN (posterior, 3.7–5.0 mm from the bregma) and NTS (posterior, 13.0–15.0 mm) areas.

#### Anatomical connections between the TMN and NTS

To confirm whether the TMN neurons directly project to the NTS, a retrograde tracer, Fluoro‐Gold (hydroxystilbamidine, Biotium, Inc. Hayward, CA) was used. Study rats (*n* = 6) were anesthetized with i.p. pentobarbital sodium (50 mg/kg), placed in a stereotaxic holder, and the dorsal surface of medulla was exposed as previously described. Fluoro‐Gold was unilaterally microinjected into the NTS (concentration, 1% in H_2_O; injection volume, 50 nL; 0.5 mm rostral to the calamus scriptorius, 0.4 mm lateral from the midline, and at a depth of 0.5 mm from the dorsal surface of the brainstem). Animals were then returned to their cages for recovery period of 7 days. Following this, 40‐*μ*m‐thick brain sections from killed animals were made. The sections were imaged using a fluorescence microscope (CKX41, Olympus, Japan) as previously described.

### Statistical analysis

All values are expressed as mean ± standard error for each group. Comparisons between two groups/conditions were made using Student's paired or unpaired *t*‐test. The criterion for statistical significance was set at *P* < 0.05.

## Results

### Effects of bicuculline microinjection into the TMN on AP and HR

**Figure 1:**
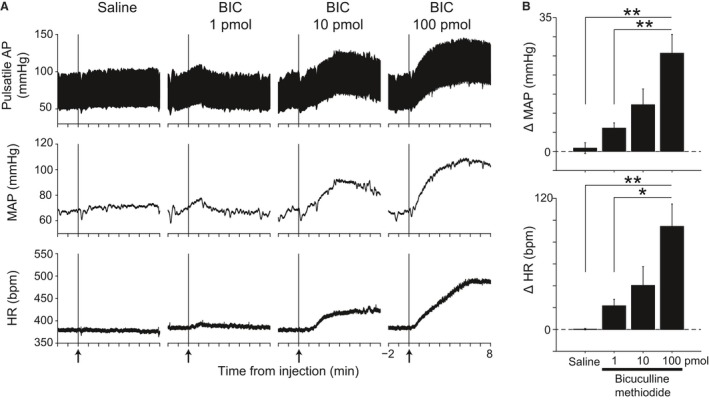
Cardiovascular changes induced by bicuculline, a GABA_A_ receptor antagonist, microinjected into the ventral part of the TMN. Representative recordings illustrating the cardiovascular changes induced by bicuculline (BIC, 1, 10, and 100 pmol/50 nL) unilaterally microinjected into the ventral TMN (A). Bicuculline microinjection elicited increases in pulsatile AP, MAP, and HR. Group data show the dose‐dependent increase in both MAP (B, top) and HR (B, bottom) in response to bicuculline microinjection (1 pmol: *n* = 5; 10 pmol: *n* = 5; 100 pmol: *n* = 7). Saline was used as a control (*n* = 4). *P < 0.05, **P < 0.01. TMN, tuberomammillary nucleus; AP, arterial pressure; MAP, mean arterial pressure; HR, heart rate.

Unilateral microinjection of bicuculline into the ventral TMN was accompanied by a dose‐dependent increase in AP as well as HR (Fig. 1). Average mean AP (MAP) following the highest dose of bicuculline administered was 95.5 ± 4.7 mmHg and was significantly higher than the baseline level (69.8 ± 2.2 mmHg, *P* < 0.01, *n* = 7). HR was 374 ± 7 bpm, and 469 ± 27 bpm (*P* < 0.01, *n* = 7) before and after stimulation, respectively. On the other hand, saline microinjection into the TMN did not affect the cardiovascular parameters (MAP, before: 75.9 ± 6.6 mmHg; after: 76.8 ± 6.3 mmHg; n.s., *n* = 4; HR, before: 379 ± 3 bpm; after: 379 ± 4 bpm; n.s., *n* = 4).

### Effects of TMN stimulation on AP and HR

**Figure 2:**
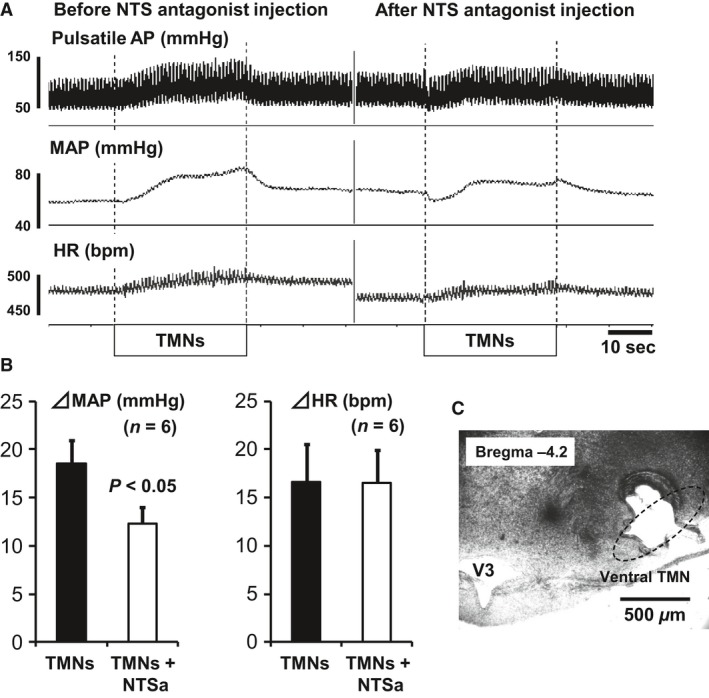
Cardiovascular changes elicited by electrical stimulation of the ventral TMN and inhibitory effects of cetirizine (H_1_ receptor‐specific antagonist) microinjected into the NTS. The ventral TMN was electrically stimulated by a microelectrode in anesthetized rats. Similar to the cardiovascular responses evoked by bicuculline microinjections into the VTM (see Fig. 1), pressor and tachycardic responses were observed (A, left). The pressor responses were partially inhibited by cetirizine (100 pmol/50 nL) microinjected into the NTS (A, right). Group data show the inhibitory effects of cetirizine on TMN stimulation‐induced pressor response but not in the tachycardic response (B). The lesion of the ventral TMN shows where the microelectrode was located (C). V3, third ventricle. TMNs, TMN stimulation. NTSa, NTS antagonist injection

Stimulation of the ventral TMN resulted in an increase in AP as well as HR (Fig. 2). Average MAP for the whole set before stimulation was 77.3 ± 7.5 mmHg, whereas the maximum MAP was 95.8 ± 5.7 mmHg (*P* < 0.001, *n* = 6). HR was 469 ± 26 bpm and 486 ± 22 bpm (*P* < 0.01, *n* = 6) before and after stimulation, respectively.

### Effects of H_1_ receptor blockade in the NTS on TMN stimulation‐induced cardiovascular responses

Microinjection of cetirizine, an H_1_ receptor antagonist, or saline into the NTS did not affect cardiovascular parameters (data not shown). However, the pressor response induced by TMN stimulation was partially inhibited when cetirizine was microinjected into the NTS (Fig. 2). In contrast, we failed to see a similar inhibitory effect on the HR response. Group data in Figure 2B demonstrate that average AP was decreased by 34% following administration of an H_1_ receptor antagonist, whereas there were no changes in HR. These results suggest that the TMN–NTS pathway is partially involved in the central pressor responses modulated by the activation of H_1_ receptors in the NTS. Saline injection into the NTS showed no effect on AP or HR responses induced by TMN stimulation (data not shown).

### Anatomical connections between the TMN and NTS

**Figure 3:**
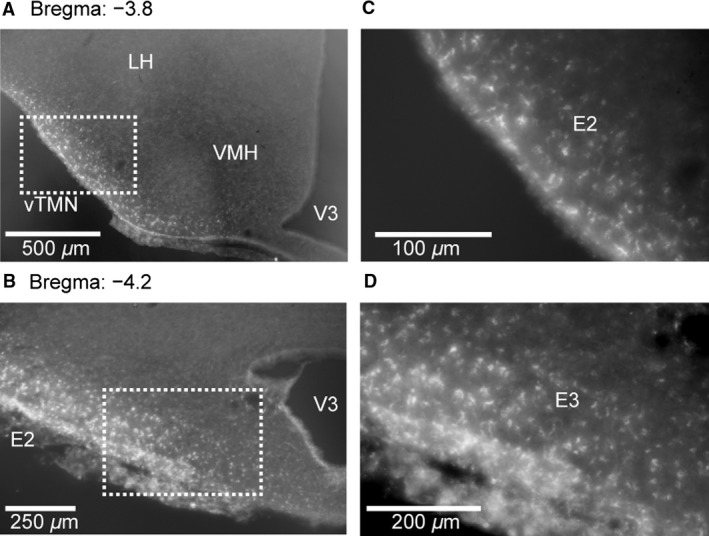
Neuroanatomical connections between the TMN and NTS confirmed by a retrograde tracer, Fluoro‐Gold. Fluoro‐Gold microinjected in the NTS was found in specific areas of the TMN in the posterior hypothalamic region (A, bregma −3.8 mm; B, bregma −4.2 mm; C, a magnified image of the dotted square in [A]; D, a magnified image of the dotted square in [B]) demonstrating a direct projection from the ventral TMN to the NTS. These areas in the TMN are known to contain histidine decarboxylase (HDC)‐positive neurons (E2 and E3, i.e., histaminergic neurons, see text). V3, third ventricle; LH, lateral hypothalamus; VMH, ventromedial hypothalamus; vTMN, ventral tuberomammillary nucleus.

Fluoro‐Gold unilaterally microinjected in the NTS was detected after a period of 7 days in the bilateral ventral TMN of the posterior hypothalamic region, referred to as E2 and E3 according to the nomenclature proposed by Wada et al. (1991) (Fig. 3). These areas are known to contain histidine decarboxylase (HDC)‐positive neurons (i.e., histaminergic neurons). These findings demonstrate that the ventral TMN neurons project directly into the NTS.

### c‐Fos expression in the TMN and NTS after exercise

**Figure 4:**
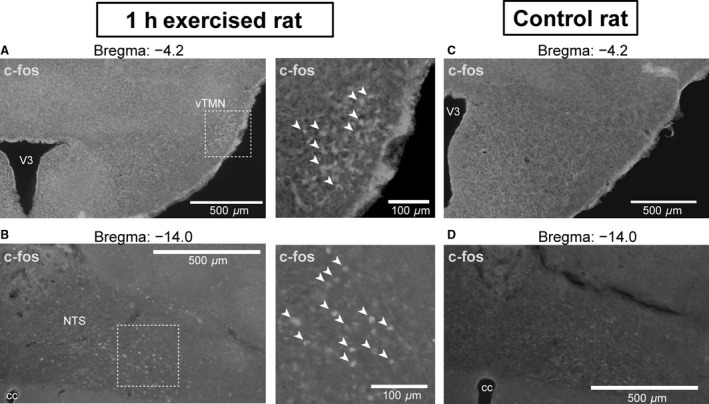
c‐Fos expression in the TMN and NTS after treadmill running test. c‐Fos‐positive cells were identified in the ventral TMN (A) and the NTS (B) after physical activity on the treadmill running test (arrowhead in right panels). The c‐Fos immunoreactivity in the TMN and NTS was much less in the no‐exercise group (C TMN; D, NTS), suggesting that these nuclei are activated by a single bout of exercise. V3, third ventricle; cc, central canal; vTMN, ventral tuberomammillary nucleus; NTS, nucleus tractus solitarius.

The expression of c‐Fos was visualized immunohistochemically in brain sections of rats subjected to the treadmill running test (Fig. 4). We found c‐Fos‐positive cells in the ventral TMN, an area where previously HDC‐positive neurons have been identified (E2) (Wada et al. 1991; Takagishi et al. 2014). Moreover, c‐Fos expression was also seen in the NTS (14.0 mm caudal to the Bregma) of animals who had been in the exercise group of the treadmill running test (Fig. 4). This area of NTS is involved in the cardiovascular function. Many studies have previously reported that microinjections of l‐glutamate induce a pressor response (Sapru 2004; Bhuiyan et al. 2011). In comparison, c‐Fos immunoreactivity in the TMN and NTS was much lesser in the no‐exercise group, suggesting that these nuclei were activated by a single bout of exercise.

## Discussion

In this study, we investigated whether the TMN–NTS pathway modulates the central cardiovascular regulation via the histaminergic system. Microinjections of bicuculline into the ventral TMN and electrical stimulation of the TMN induced pressor and tachycardic responses. The pressor responses were partially inhibited by cetirizine microinjected into the NTS, whereas no such inhibitory effects were observed on the HR responses. Using histological methods, we also confirmed the projection of histaminergic neurons from the ventral TMN neurons into the NTS. In addition, we also demonstrated that a single bout of exercise could elevate c‐Fos expression in both the TMN and NTS neurons. These results suggest that the TMN–NTS pathway modulates AP, and this may partially explain exercise‐induced AP responses.

Histaminergic neurons originating from the TMN are important regulators of the sleep–wake cycle (Haas and Panula 2003; Haas et al. 2008). They are active during the arousal phase, whereas during the sleep phase they are inhibited via a GABAergic input that arises from the hypothalamic ventrolateral preoptic area (Sherin et al. 1998; Haas et al. 2008). Some general anesthetics are known to act on GABAergic afferents to the TMN nucleus (Nelson et al. 2002; Sergeeva et al. 2005). Therefore, we microinjected bicuculline into the TMN to inhibit the GABAergic effects on TMN neurons, and as a result, found pressor and tachycardic effects similar to those induced by electrical stimulation of the TMN. These results suggest that TMN neurons may be involved in increasing AP and HR during arousal phase. Because of technical limitations, primarily the difference in time resolution (i.e., stimulation must occur during the period that the H_1_ receptor antagonist takes effect), we chose electrical stimulation instead of drug microinjections into the ventral TMN to functionally identify a neuronal pathway between the TMN neurons to NTS. Thus, we cannot rule out the possibility that cardiovascular responses induced by TMN electrical stimulation are partially mediated by activation of passing nerves through the TMN. However, these results suggest that, at least partially, the TMN–NTS pathway regulates AP increase via histaminergic modulation.

**Figure 5:**
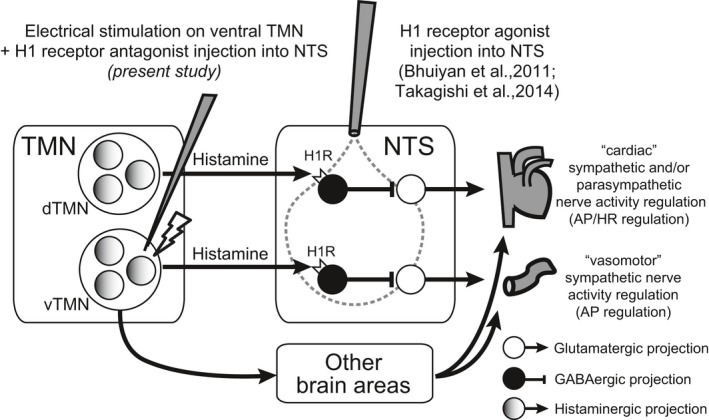
A schematic illustration of the presumptive pathway between TMN histamine projections to NTS and its effects on cardiovascular regulation. Microinjection of histamine H_1_ receptor antagonist partially prevented the pressor effect but had no effect on heart rate in response to electrical stimulation of the ventral TMN (Fig. 2). This is because ventral TMN neurons mainly modulate NTS neurons that control “vasomotor” sympathetic outflow. Electrical stimulation of the ventral TMN evokes histamine release from synaptic terminals and activates NTS neurons with histamine receptors that receive ventral TMN inputs. In addition, electrical stimulation of the ventral TMN not only directly activates the NTS projection pathway but also indirectly and/or independently activates through the other brain areas that control both “vasomotor” sympathetic and “cardiac” sympathetic and/or parasympathetic nerve activities. NTS, nucleus tractus solitarius; TMN, tuberomammillary nucleus.

Neurons in the NTS, which respond to baroreceptor inputs (i.e., barosensitive neurons), excite GABAergic inhibitory neurons that project from the caudal ventrolateral medulla to the rostral ventrolateral medulla (RVLM) and inhibit the glutamatergic neurons there, resulting in decreased sympathetic preganglionic neuronal outflow (Sapru 2004). Thus, our results suggest that the TMN–NTS histaminergic pathway increases blood pressure by inhibiting NTS barosensitive neurons. Considering that the H_1_ receptor is a member of the G protein‐coupled receptor superfamily and that excites neurons in most brain regions through activation of the Gq/11‐phospholipase C pathway (Haas et al. 2008), it follows that histamine release in NTS could activate local inhibitory interneurons, thereby inhibiting barosensitive NTS neurons and evoking a net sympathoexcitation. Alternatively, H_1_ receptor‐expressing neurons in the NTS could be chemosensitive and directly activate sympathetic premotor neurons in the RVLM to increase AP, although this hypothesis remains to be tested. An interesting observation from our study was that cetirizine microinjected into the NTS did not inhibit TMN stimulation‐induced tachycardia, suggesting TMN neurons mainly modulate NTS neurons that control vasomotor sympathetic outflow and not the cardiac parasympathetic and/or sympathetic outflow. This result appears to contradict our previous observation in which exogenous administration of an H_1_ receptor agonist into NTS produced an increase in AP and HR (Bhuiyan et al. 2011). We believe these counterintuitive observations may be because of at least two reasons. First, H_1_ receptor agonist injection into NTS activates neurons with H_1_ receptor receiving inputs not only from the ventral TMN but also from other regions, whereas electrical stimulation of ventral TMN evokes histamine release from the synaptic terminals of NTS‐projecting ventral TMN neurons and causes activation of NTS neurons with histamine H_1_ receptors exclusively receiving ventral TMN inputs. Second, the TMN neurons are able to increase both AP and HR without mediating NTS neurons because electrical stimulation of TMN stimulates not only the TMN–NTS pathway but also pathways from TMN to other brain areas (Fig. 5).

Questions arise, such as *which physiological condition is related to the TMN–NTS pathway*? Since we previously found that the pressor response but not tachycardia mediated by activated H_1_ receptors in the NTS was increased after long‐term daily exercise (Waki et al. 2013), histamine and H_1_ receptors in the NTS are likely to be involved in exercise‐related AP regulation. In addition to this, we found a high level of c‐Fos expression in both the ventral TMN and NTS after the treadmill running test in our study, suggesting potential involvement of these areas of the brain during a single bout of exercise. It is generally accepted that physical activity, such as running, induces an increase in AP and HR mainly due to sympathoexcitation (Ludbrook and Graham 1985; Miki et al. 2003; Matsukawa 2012; Waki 2012). The current understanding of brain mechanisms involves a feedforward (i.e., central command) process that originates from motor control‐related areas, such as the insular cortex, and hypothalamic and mesencephalic locomotor regions (Goodwin et al. 1972; Williamson et al. 2003; Matsukawa 2012; Liang et al. 2016). Accumulating evidence suggests that the NTS is one of the key nuclei involved in the central command mechanism (Michelini and Stern 2009; Waki 2012; Waki et al. 2013). We hypothesized that histaminergic neurons in the ventral TMN may also be involved in the central command regulation of AP via modulation of NTS functions. Some c‐Fos‐positive neurons in the NTS found after a single bout of exercise could be GABAergic interneurons that inhibit the NTS barosensitive neurons, thus allowing AP to rise.

In summary, our findings suggest that the TMN–NTS pathway is most likely to be involved in the central regulation of blood pressure and may have an important role in regulation of the cardiovascular system under high‐arousal states, such as during physical activity.

## Conflict of Interest

None declared.
